# Live video footage from scene to aid helicopter emergency medical service dispatch: a feasibility study

**DOI:** 10.1186/s13049-019-0632-4

**Published:** 2019-05-08

**Authors:** E. ter Avest, E. Lambert, R. de Coverly, H. Tucker, J. Griggs, M. H. Wilson, A. Ghorbangholi, J. Williams, R. M. Lyon

**Affiliations:** 1 Air Ambulance Kent, Surrey and Sussex, Redhill Aerodrome, Redhill, Surrey RH1 5YP UK; 2 Department of Emergency Medicine, University Hospital Groningen, Groningen, the Netherlands; 3 School of Health Sciences, University of Surrey, Guildford, UK; 4 Neurotrauma Centre, Imperial College, London, UK; 5 GoodSAM, 1 CurtainRd, London, UK; 6 South East Coast Ambulance Service NHS Foundation Trust, Crawley, UK

**Keywords:** Helicopter emergency medical services (HEMS), Dispatch, Video, Trauma

## Abstract

**Background:**

Obtaining accurate information from a 112 caller is key to correct tasking of Helicopter Emergency Medical Services (HEMS). Being able to view the incident scene via video from a mobile phone may assist HEMS dispatch by providing more accurate information such as mechanism of injury and/or injuries sustained. The objective of this study is to describe the acceptability and feasibility of using live video footage from the mobile phone of a 112 caller as an HEMS dispatch aid.

**Methods:**

Live footage is obtained via the 112 caller’s mobile phone camera through the secure GoodSAM app’s Instant-on-scene™ platform. Video footage is streamed directly to the dispatcher, and not stored. During the feasibility trial period, dispatchers noted the purpose for which they used the footage and rated ease of use and any technical- and operational issues they encountered. A subjective assessment of caller acceptance to use video was conducted.

**Results:**

Video footage from scene was attempted for 21 emergency calls. The leading reasons listed by the dispatchers to use live footage were to directly assess the patient (18/21) and to obtain information about the mechanism of injury and the scene (11/21). HEMS dispatchers rated the ease of use with a 4.95 on a 5-point scale (range 4–5). All callers gave permission to stream from their telephone camera. Video footage from scene was successfully obtained in 19 calls, and was used by the dispatcher as an aid to send (5) or stand down (14) a Helicopter Emergency Medical Services team.

**Conclusion:**

Live video footage from a 112 caller can be used to provide dispatchers with more information from the scene of an incident and the clinical condition of the patient(s). The use of mobile phone video was readily accepted by the 112 caller and the technology robust. Further research is warranted to assess the impact video from scene could have on HEMS dispatching.

## Background

Major trauma is a leading cause of mortality and serious morbidity, especially in the young. Helicopter Emergency Medical Services (HEMS) attend the most severely injured trauma patients. For these patients, time is critical to save life and to prevent long-term disability [1–3]. Accurate and early dispatch is therefore paramount. Careful selection of patients who might benefit from HEMS interventions is important, as HEMS resources are limited [4], and being tasked to an incident that does not benefit from HEMS interventions may prevent a HEMS response at a concurrent incident that does.

It is the HEMS-dispatchers’ role to obtain crucial information about the scene, the mechanism of injury and the clinical state of the patient(s) in a timely manner, in order to decide whether a HEMS team should be dispatched or not. This is challenging, as bystanders making an emergency call are usually not medically trained, and are often emotionally affected by what they witness. This can make it hard for them to describe complex scenes and/or the clinical condition of the patient(s) [5]. Description of a clinical condition, such as conscious level or respiratory pattern, over the telephone can be challenging. As a result, prolonged interrogation of a 112 call is sometimes necessary, with limited (and sometimes inaccurate) information being provided about the patient’s condition. This may result in a delayed or inaccurate dispatch. Previous studies have shown that mechanism of injury together with interrogation by the dispatcher had a sensitivity of 80.2% and under-triage of 19.7% for the identification of major trauma [6, 7], and a Dutch study showed an overall over-triage of 44% [8].

The ability of a HEMS dispatcher and HEMS response team to view the scene in real time, at the point of the emergency call, could allow more accurate scene assessment and more appropriate, timely dispatch of emergency medical services, including HEMS. For this purpose, live video stream from scene is currently being trialed by Air Ambulance Kent Surrey and Sussex (AAKSS), allowing the AAKSS dispatcher to speak to the caller of the emergency number and ask whether they are in a position to stream live video footage from their mobile phone to the dispatcher in the control room.

The objective of this study is to describe the acceptability and feasibility of the use of live video footage as a dispatch aid for HEMS dispatchers.

## Methods

### Setting

This is a prospective study of all calls made to the emergency number (112/999) evaluated by the HEMS dispatchers of the Air Ambulance Kent, Surrey and Sussex between March and December 2018. AAKSS is a Helicopter Emergency Medical Service (HEMS) covering three counties in the southeast of England with a resident population of 4.5 million and transient population of up to 8 million. Two doctor/paramedic teams respond 24/7 in either a helicopter or response car, attending approximately 2500 missions per year. Statistics from the UK National Audit Office suggest that in this region of the UK, there are approximately 630 cases of major trauma annually.

### HEMS dispatch protocol

The AAKSS HEMS team (consisting of two pilots, a paramedic and a doctor) is dispatched by a dedicated AAKSS dispatcher who is present in the South East Coast Ambulance Service (SECAmb) control room, and continuously screens incoming emergency calls. AAKSS dispatchers have a background of ambulance dispatch, with extensive experience of working in the ambulance control room. As part of their HEMS dispatch training they are put through an induction course, followed by a four-week development period, starting with observation of the dispatch desk progressing to peer supervised practice and culminating in a sign-off assessment undertaken by an operational manager. All dispatchers observe regularly on operational shifts to improve awareness, and participate in the AAKSS (ongoing) internal audit process. Dispatchers are aided by a bespoke tasking algorithm, devised by the AAKSS management team [9]. Whilst listening to the incoming emergency call, dispatchers aim to rapidly identify either one (from Grade 1 criteria list) or two (from Grade 2 criteria list) dispatch criteria [9]. If these are positively identified, HEMS is dispatched.

### Video footage

**Fig. 1:**
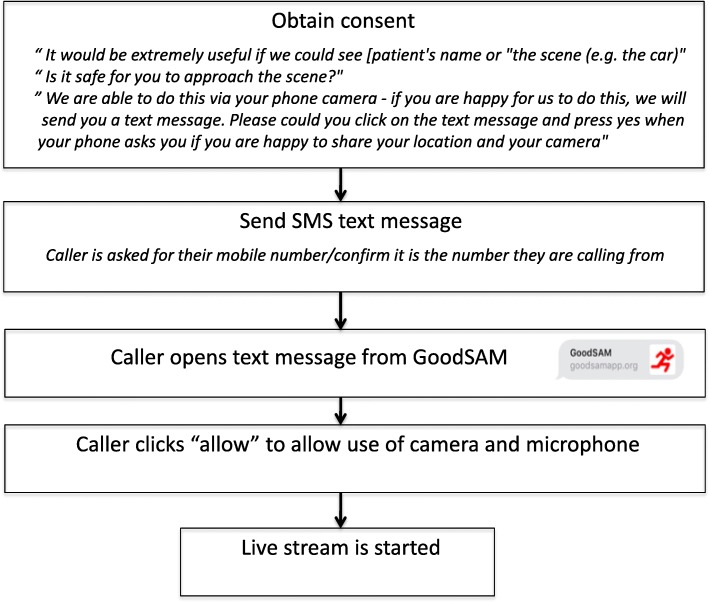
Process of obtaining live video from scene

Video footage is streamed from scene to the control room using the GoodSAM (Smartphone Activated Medics; www.goodsamapp.org) platform, a globally used tool to task first responders to suspected out-of-hospital cardiac arrests [10]. The GoodSAM Instant-on-Scene function™ provides the HEMS dispatcher with the ability to request the caller of the emergency number to activate the video camera on their mobile phone and securely stream live video footage in real time to the control room. The caller does not require any App (such as FaceTime™ or Skype™), only a video-capable mobile phone. The steps to obtain live video are described in Fig. 1. When the dispatcher is logged into the GoodSAM platform and an emergency call comes in, the caller is first asked if it is safe for them to approach the scene. Subsequently, permission is asked to obtain access to the caller’s phone camera, and an SMS text message is sent, asking the caller to confirm their consent to share their location and stream video from their mobile phone camera. When the caller confirms consent, the mobile phone automatically starts securely transmitting a video live stream from the scene directly back to the HEMS dispatcher in the ambulance control room. The footage is not recorded or stored, either on the mobile phone or on the GoodSAM portal. The live stream is maintained until cancelled by the dispatcher. Currently, video footage can be used to assess the scene, the mechanism of injury and the clinical condition of the patient. Objective assessment of vital signs such as pulse rate from the video feed also occurs but was not analyzed in this study.

### Outcome measures

This pilot study focused on acceptability and feasibility of the use of video footage from scene. Outcome measures were defined as:

1. Acceptability for emergency callers to use video transmission from scene.

2. Feasibility of the HEMS dispatch system to use video footage from scene as an aid in HEMS dispatch.

### Data acquisition

HEMS dispatchers were instructed on the use of the GoodSAM Instant-on-Scene™ platform prior to the study period. They could use the video footage at their discretion for all emergency calls during the study period as an extra tool before deciding on dispatch. Use of the system had no influence on normal ambulance service (SECAmb) emergency call handing or ambulance dispatch. When video footage was used, HEMS dispatch was in accordance with the normal protocol assisted by the footage.

Each time the dispatchers used live video footage from scene, the following characteristics were recorded by the dispatcher after the call: Ambulance call number, reason for use of video triage, type of individual asked to stream from scene (layperson, patient, professional), acceptability for the caller to use their camera to obtain footage as judged by the dispatcher (0–5), ease of use by dispatcher (0–5), specific variables around ease of use, any technical- or user issues, and the result of the use of the video footage (dispatch or no dispatch of HEMS team).

### Ethics

Video footage was obtained under UK CCTV regulations for use. Callers were informed of the purpose of streaming live footage (to aid and improve triage decisions and clinical care for the patient). Neither the video nor audio footage was recorded on the caller’s telephone or the receiving computer, as they were streamed on the Instant-on-scene™ app platform, which is confidential and compliant with the UK data protection act [11]. This project met National Institute for Healthcare Research (NIHR, UK) criteria for service evaluation and formal ethical approval was therefore not required. The project was approved by the AAKSS Research & Development Committee and the South East Coast Ambulance Service NHS foundation trust (SECAmb) Research and Development Group.

## Results

### Call characteristics

During the study period, video footage from scene was obtained by the HEMS dispatchers for 21 emergency calls. The commonest reasons listed by the dispatchers to use the system were to assess the patient (18/21) and to obtain information about the mechanism of injury (MOI) and the scene (11/21). Most often a member of public was asked to stream from scene, although in a minority of cases the police (2) or an off-duty nurse (1) was asked to do so. In two cases patients themselves were asked to provide video footage (Table 1).

**Table 1:** Acceptability and feasibility of obtaining live video footage from scene using the GoodSAM ““

Job No	Reason for using video triage	What individual asked to stream from scene	How do you feel the caller accepted use of GoodSAM?	Ease of use by dispatcher	Technical issues	User issues	Intervention after watching video stream.
	(to assess patient, scene / MOI, or other)	(member of public, police, FRS, healthcare professional, patient)	0 = poor 1- mediocre 2 = acceptable 3 = good 4 = very good 5 = excellent	0 = poor 1 = mediocre 2 = acceptable 3 = good 4 = very good 5 = excellent			
1	Patient/MOI	Police	5	5	Poor video quality	No issue	Confirmed send
2	Patient/MOI	Police/public	5	5		No issue	Confirmed send
3	Patient/MOI	Public	5	5		No issue	Confirmed send
4	Patient/MOI	Public	5	5		No issue	No send
5	MOI	Public	5	5		No issue	Confirmed send
6	Patient	Public	5	5		No issue	No send
7	Patient	Public	5	5		No issue	No send
8	Patient/scene	Public	5	5		No issue	No send
9	MOI/scene	Public	5	5		No issue	No send
10	Patient/ MOI	Public	5	5		Caller closed call	Confirmed send
11	Patient		4	5		No issue	No Send
12	Patient	Public	5	5		No Issue	No Send
13	Patient	Public	5	5		No Issue	No Send
14	Patient	Patient	5	5		No Issue	No Send
15	Patient	Patient	5	5		No Issue	No send
16	MOI	Public	5	5		No issue	No Send
17	Patient	Public	5	4	Inability to stream		N/A
18	Patient	Public	5	5		No Issue	No Send
19	Patient/MOI	Public	5	5		No Issue	No Send
20	Patient	Public	5	5	Inability to stream	No issue	N/A
21	Patient/MOI	Public	5	5	Poor audio quality	No issue	No send

### Acceptability and ease of use

Overall, the HEMS dispatchers rated the ease of use of the GoodSAM Instant-on-scene™ platform with a mean of 4.95 on a 5-point scale (range 4–5). All callers who were asked permission to stream from their telephone camera were willing to do so. No caller refused to transmit live footage. All callers understood the instructions given by the dispatcher and received the SMS asking them their consent to share both their location and their phone camera. Video footage from scene was obtained for 19/21 calls. In two calls there was no data coverage, precluding video streaming from scene. One caller accidentally closed the call but continued streaming, and was called back by the dispatcher. The quality of the video stream was rated as good in 18/19 calls, and poor in 1/19. In one call there were audio issues, but this did not preclude the use of the footage as a dispatch aid.

### Results of using on scene video

After watching the footage from scene, the dispatchers used the footage to confirm sending the HEMS team in 5/19 calls, whereas they decided not to send the team in the remaining 14 calls.

## Discussion

In this pilot study, we demonstrated that live video footage from scene can be used to provide dispatchers with more information about the scene of an incident and the clinical condition of the patient(s), thereby assisting the HEMS tasking decision.

We found that the platform used to obtain live video footage, the GoodSAM Instant-on-scene™, was easy to use for both dispatcher and caller, and was reliable in providing the desired footage, with technical issues occurring only in a small minority of the emergency calls it was used in. As the platform does not require installation of any specific apps on the callers’ phone, it can be used by almost anybody with a mobile phone, which means that the potential availability is considerable. Our data show that the willingness of the public to help by allowing access to their phone camera in order to stream video footage was high. In this regard it is important to mention that the caller was made aware of the fact that video footage is not stored on either end, and that the dispatcher cannot access any data on the caller’s phone. Furthermore, it should be noted that this study was carried out in a non-urban area. Willingness- and ability of callers to help may be lower in more urban areas, for example through the presence of language barriers.

Since a bespoke tasking algorithm was used by our dispatchers [9], it was anticipated that the video footage from scene would be used in only a minority of the emergency calls made (21 during the study period). When dispatch criteria were already met during the emergency call, the HEMS team was dispatched instantly. Only when the criteria of the tasking algorithm were not (yet) met, video footage was considered as an additional aid. In those instances the dispatchers found it extremely helpful to visualize the patient or the scene to get a better impression of the mechanism of injury or the injuries sustained. Members of the public are not trained to provide this information, and it is sometimes a difficult and lengthy process for the dispatchers to obtain the right information during a phone call. What might seem serious injuries to a lay person may not justify sending a HEMS team, whereas sometimes injuries are described to be minor, with the patient actually needing a critical intervention only a little later [12]. Furthermore, often multiple calls are made to the emergency number from the same incident scene, with different callers sometimes providing conflicting information to the dispatcher. Finally, language barriers may be present, preventing a quick description of the scene and injuries [13]. In these instances, being able to view the scene and the patient(s)’ clinical condition can make a big difference to the dispatcher.

In this initial feasibility study, video footage was used as a dispatch aid in 19 calls. In 5 instances, the dispatcher decided to send the HEMS team, whereas in 14 the HEMS team was not sent. It was beyond the scope of this feasibility study to determine whether the video footage contributed to a correct dispatch of HEMS. Although there are potential advantages of using live footage (more accurate dispatch), there may also be potential disadvantages. First, it is unclear what the influence of using live footage is on dispatch timings. Although the live video stream was established instantly in 19/21 cases in our study, it was not recorded how long streaming continued before the decision was made (not) to dispatch. Therefore, it is unclear how the dispatch times compares to the average (daytime) dispatch time of 10 min for the dispatches in the study period where live video footage was *not* used. Future studies are needed to investigate the contributive value of using live video footage and the effect it has on dispatch times. Furthermore, it is important to realize that when using live video footage as a dispatch aid, dispatchers and members of the public are asked to film and witness potentially shocking scenes they are not familiar with. So far, dispatchers have not experienced the snapshots of the scenes they witnessed as shocking. However, adequate support should be available to dispatchers, and offered to callers as well when needed. Finally, as the footage was used during daytime dispatches only in our study, we have no information yet about the quality of the video footage in more sparsely lit circumstances.

## Conclusion

Live video footage from scene is an acceptable and feasible aid for HEMS dispatch. Further studies are needed to demonstrate its merit in improving HEMS dispatch accuracy and exploring potential for use more widely across ambulance services.

## Abbreviations

AAKSS: Air Ambulance Kent Surrey and Sussex
GoodSAM: GoodSAM (Smartphone Activated Medics)
HEMS: Helicopter emergency Medical service
MOI: Mechanism of injury
